# Isolation and sequencing of a novel inovirus, “Copypasta,” from Rhine River water

**DOI:** 10.1128/mra.01180-23

**Published:** 2024-01-24

**Authors:** P. Nathael Javorčík, Alexander Harms

**Affiliations:** 1 Institute of Food, Nutrition and Health, D-HEST, ETH Zurich, Zurich, Switzerland; 2 Biozentrum, University of Basel, Basel, Switzerland; Queens College, Queens, New York, USA

**Keywords:** filamentous bacteriophage, inovirus, *Escherichia coli*

## Abstract

We present a new inovirus named Copypasta isolated from the Rhine River that infects *Escherichia coli* and shows the expected filamentous morphology. Copypasta has a circular single-stranded DNA genome that is 6,408 nt long and harbors 12 protein-coding genes.

## ANNOUNCEMENT

Bacteriophages are the most abundant biological entities on Earth and display large genomic and morphological diversity (1). Most characterized phages belong to the Caudoviricites class of tailed phages with double-stranded DNA genomes (2). Conversely, inoviruses are distinct non-tailed phages with characteristic filamentous virions, typically between 1 and 2 µm long (3, 4). Representatives infecting *Escherichia coli* K-12 like M13 or f1 have been well studied and use the sex pilus of the conjugative F-plasmid as host receptor (3–5). These viruses replicate inside host cells and are then secreted without lysis (6). The circular single-stranded DNA genomes of inoviruses are between 4 and 12 kb long, encoding around 10 proteins for virion structure, assembly, and secretion (4, 7). Among these, the minor and major capsid proteins are known as excellent platforms for phage display (3, 8). Despite the abundance and diversity of inoviruses in prokaryotic genomes and metagenomes (9), only few inoviruses have been isolated and cultured.

**Fig 1:**
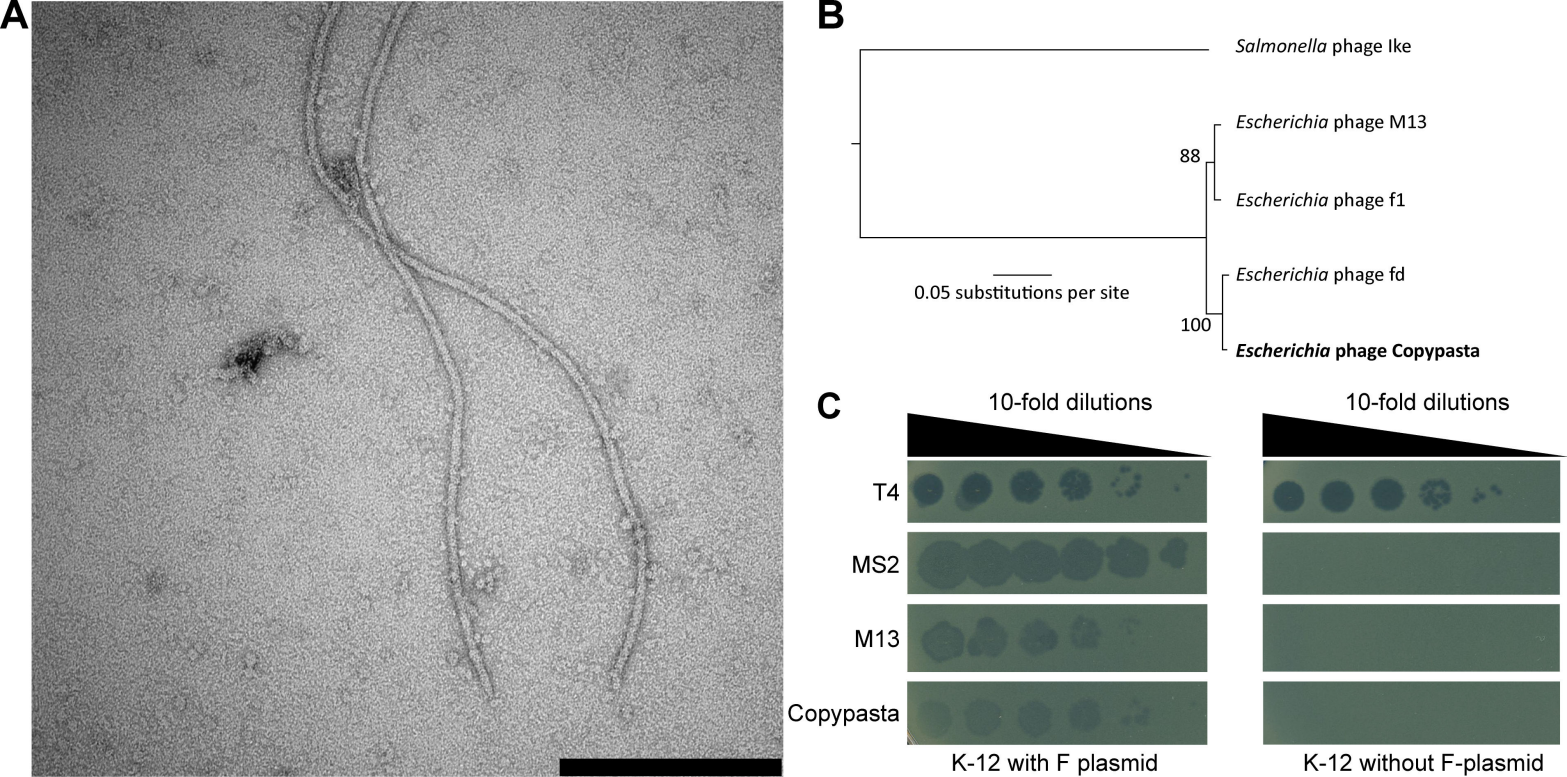
Copypasta is a novel inovirus isolated from the Rhine River. (**A**) A negative-stain transmission electron microscopy image shows Copypasta’s filamentous morphology (black bar = 200 nm). (**B**) The whole-genome maximum-likelihood phylogeny shows a close relationship of Copypasta (bold) with other Ff phages. *Salmonella* phage Ike was included as an outgroup to root the tree. A sequence alignment had been generated using MAFFT v.7.490 (11) implemented in Geneious Prime v.2023.2.1 using default settings and was then used to calculate the phylogeny using PhyML v.3.3.20180621 (12) implemented in Geneious Prime v.2023.2.1 with the HYK85 substitution model and default settings. Branch numbers show bootstrap support. (**C**) The soft-agar spotting assay shows the dependence of Copypasta and, as shown previously, closely related inovirus M13 and *Leviviridae* phage MS2 on the F-plasmid sex pilus as a host receptor (3, 13). Phage T4 was included as a control that is not affected by the presence or absence of an F-plasmid.

Here we report the isolation and characterization of Copypasta, a new inovirus infecting *E. coli*. Copypasta was isolated from Rhine River in Basel, Switzerland (coordinates 47°33′24.9″N 7°35′57.3″E, 2 July 2019), using an *E. coli* K-12 strain with F-plasmid as host. The isolation was done by direct plating of concentrated water samples on soft-agar overlays as described previously (10). Briefly, the host was cultured in lysogeny broth (LB) medium at 37°C; samples were concentrated using ZnCl_2_ precipitation, and the selected isolate was restreaked three times from single plaques to ensure clonality. Negative-stain transmission electron microscopy revealed the expected filamentous morphology of Copypasta virions (Fig. 1A).

For sequencing, genomic DNA was prepared from 1 mL of a high-titer phage stock using a commercial kit (Norgen Biotek, Thorold, ONT, Canada). The genome was sequenced at MiGS (Microbial Genome Sequencing Center, Pittsburgh, PA, USA) on an Illumina NextSeq 2000 platform producing 2 × 151 bp reads from libraries prepared using bead-based transposome fragmentation and PCR amplification. Adapters had been removed from sequencing reads by MiGS. Of 3,230,050 reads in total, we used 15,271 reads to assemble the genome of Copypasta with Geneious’ in-built assembly function (Geneious Prime v.2023.2.1). Before this, the assembler trimmed the reads with an error probability limit of 0.1 at the 5′ and 3′ ends. The assembly resulted in one circular contig of 6,408 nt with a GC content of 40% and a mean coverage of 298.8×. The genome was annotated using pharokka v.1.2.0 (14), which identified 12 protein-coding genes. A whole-genome alignment using MAFFT v.7.490 (11) implemented in Geneious Prime v.2023.2.1 showed a close relationship of Copypasta with well-studied phages f1, fd, and M13, collectively known as Ff phages (15), which have >96% nucleotide sequence identity among each other and with Copypasta (Fig. 1B). Copypasta grows well on *E. coli* carrying the conjugative F-plasmid but cannot infect strains without it (Fig. 1C). This result confirms the sex pilus as host receptor of Copypasta like for other Ff phages (3, 4). Further analyses of Copypasta may elucidate novel mechanisms of virus-host interactions and provide novel platforms for phage display.

## Data Availability

The genome sequence and relevant metadata of phage Copypasta have been deposited in National Center for Biotechnology Information (NCBI) GenBank under accession number OR791786, BioProject number PRJNA1040766, and BioSample number SAMN38264365. Raw reads that have been used for the assembly of the genome are available in the NCBI Sequence Read Archive under accession number PRJNA1040766.
